# Increased abundance of a common scavenger affects allocation of carrion but not efficiency of carcass removal in the Fukushima Exclusion Zone

**DOI:** 10.1038/s41598-022-12921-y

**Published:** 2022-05-26

**Authors:** Hannah C. Gerke, Thomas G. Hinton, Kei Okuda, James C. Beasley

**Affiliations:** 1 Savannah River Ecology Laboratory and Warnell School of Forestry and Natural Resources, University of Georgia, PO Drawer E, Aiken, SC 29802 USA; 2 Institute of Environmental Radioactivity, Fukushima University, Fukushima, Japan; 3 CERAD CoE, Norwegian University of Life Sciences, Faculty for Environmental Sciences and Natural Research Management, Aas, Norway; 4 Hiroshima Shudo University, Hiroshima, Japan

**Keywords:** Ecology, Community ecology, Ecosystem services

## Abstract

The 2011 nuclear accident in Fukushima, Japan caused the evacuation of > 100,000 people and prompted studies on environmental impacts of radiological contamination. However, few researchers have explored how the human evacuation has affected ecosystem processes. Despite contamination, one common scavenger (wild boar, *Sus scrofa*) is 2–3× more abundant inside the Fukushima Exclusion Zone (FEZ). Shifts in abundance of some scavenger species can have cascading effects on ecosystems, so our objective was to investigate impacts of the evacuation and the resulting increase in wild boar on vertebrate scavenger communities. We deployed cameras at 300 carcasses in the FEZ and a nearby inhabited area, and quantified carcass fate, scavenger species, and detection/persistence times. We also tested effects of carcass size and habitat on scavenger community composition and efficiency by balancing trials across two carcass sizes and habitats in each zone. Overall scavenger richness and carcass removal rates (73%) were similar in the FEZ and inhabited area, but species-specific carcass removal rates and occurrence differed between zones. Wild boar removed substantially more carcasses inside the FEZ, with implications for nutrient and contaminant distribution. Our results suggest carcass size affects scavenging dynamics more than human activity or habitat, and abundance changes of common scavengers can influence carrion resource allocation.

## Introduction

By offering services like carrion removal, nutrient cycling, and disease control, vertebrate scavengers play a valuable role in maintaining ecosystems^1–3^. Vertebrate scavengers are an essential stabilizing force in most ecosystems because they increase the complexity of food webs by providing extra interspecies links^4–6^. A high degree of overlap within the scavenging community results in increased functional redundancy that can support food web persistence during large disturbances^7–9^. However, increased anthropogenic activities in the last several decades have caused rapid losses in global biodiversity (the ‘sixth mass extinction’), and vertebrate scavengers are no exception^3,10–13^. Processes negatively impacting scavenging communities can have resounding consequences for the stability of ecosystems and their ability to provide biologically and socioeconomically valuable services^13–15^.

In the context of biodiversity losses, identifying how to preserve the function of scavenging communities is particularly important. The richness of communities has previously been considered a key aspect of ecosystem function^8^, but recent work suggests the functional identity of species—which considers species’ specific traits—may be more influential in some ecosystem processes^16–19^. While scavenging is a common foraging strategy used by many taxa, not all scavengers are equally efficient^20^, with dominant and/or obligate scavengers such as large carnivores and vultures often removing the majority of carrion when present^21–24^. Winfree et al.^25^ suggested the abundance of the dominant/common species drives ecosystem services more than species richness alone, while Mateo-Tomás et al*.*^26^ illustrated that species richness increased carrion consumption in areas where obligate scavengers or large carnivores were rare. Similarly, Gutiérrez-Cánovas et al.^23^ found that increased carcass consumption rates were explained more by the presence of specific traits in scavenger assemblages (e.g., large home ranges and high mobility, social scavenging, large body size) than metrics like scavenger diversity or species richness.

Nonetheless, human-mediated global declines in vultures and large carnivores mean scavenging communities are increasingly dominated by facultative scavengers that are often mesocarnivores or generalists^27–31^. Scavenging dynamics in such systems may or may not achieve the same efficiency (i.e., rate of detection and/or removal), depending on the composition of the remaining community^9,30,32–34^. In particular, facultative scavenger communities without efficient avian scavengers may be more sensitive to changes in the abundance of common scavengers^35–37^. Although several studies indicate human activity can influence the distribution or activity of facultative scavenger species^38–42^, fewer have tested the actual impact on scavenging dynamics^43^. Factors like urbanization and land use can affect the composition and/or efficiency of some facultative scavenger communities^9,28,44^, but understanding the changes in scavenging dynamics resulting from anthropogenic disturbances remains a priority^2^.

The 2011 Fukushima Dai-ichi Nuclear Accident forced over 100,000 residents to evacuate from their homes, leaving a 371 km^2^ area where humans are still unable to return (officially termed the “Difficult-to-Return Zone”; referred to herein as the Fukushima Exclusion Zone, FEZ)^45^. Despite a push for radioecological research to describe the environmental impacts of radionuclide contamination, few researchers have investigated how anthropogenic aspects of the accident—such as the evacuation of humans—have affected ecosystem processes^46^. The FEZ offers a unique opportunity to study scavenging dynamics following massive shifts in human activity in a landscape where population densities, infrastructure, and land use were similar prior to the accident (26 vs. 30 people/km^2^)^46,47^. Recent work via extensive camera trapping across a gradient of human activity and radiation levels suggests that despite the presence of radioactive contamination, populations of many facultative scavenger species in the FEZ are not significantly reduced compared to those in surrounding areas^48^. In fact, some species that conflict with humans—such as wild boar (*Sus scrofa*)—are substantially more abundant inside the evacuated FEZ, and shift their activity patterns across areas of differing human presence^48^. Similar observations of increased populations of wild boar as well as other large ungulates and apex predators (e.g., gray wolves—*Canis lupus*) have been reported within the Chernobyl Exclusion Zone^49,50^, resulting in a diverse and highly efficient vertebrate scavenging community^51^.

As changes in abundance and activity of facultative scavengers have potential repercussions for scavenging dynamics and ecosystem function^52^, our primary objective was to explore the effects of human habitation on vertebrate scavenging communities in and around the FEZ. Previous studies have shown that habitat and carcass size can significantly influence scavenging dynamics^22,53^, so our secondary objectives were to test their effects on scavenger community composition and efficiency within our experimental framework. We hypothesized that large increases in the abundance of a common facultative scavenger—wild boar—due to the evacuation of humans would alter scavenging dynamics in the FEZ, resulting in higher efficiency of carcass removal compared to human inhabited areas outside the FEZ. We also expected scavenger composition to vary across habitats, and for larger carcasses to persist longer and have higher scavenger richness, but that these patterns would be consistent across our two study regions. Finally, weather-related factors such as temperature, wind speed, and humidity may affect carcass persistence by influencing scavenger activity and/or carcass detection^54–56^. Our study took place during Japan’s rainy season (June to August), so we also tested the hypothesis that rain would increase the time until carcass detection and depletion. For example, carcasses may persist longer at cooler temperatures due to suppression of invertebrate and microbial activity^20^, and weather conditions during rainfall could impair the ability of vertebrate scavengers to detect carcasses using olfactory cues^54,55^.

## Methods

### Study site

A large portion of the landscape surrounding the Fukushima Daiichi Nuclear Power Plant was evacuated after the accident in March 2011, but beginning in 2016 restrictions were lifted in portions of that area to allow human repopulation following the decrease in radiation to safe levels^45^. As of 2017, the FEZ covered approximately 371 km^2^ of land where contaminant levels remained too high for human habitation^45^, and thus had substantially less human activity compared to the surrounding landscape^57^. Although extensive decontamination efforts in the contaminated areas surrounding the FEZ were completed by March 2018, the majority of the FEZ had not been decontaminated^57,58^, with limited decontamination efforts concentrated around roads and residential areas completed between 2012 and 2016^57^. Future decontamination efforts in the FEZ will be undertaken upon request by residents planning to return in the coming decades when it is safe to do so. Our study site consisted of two sampling areas, one inside the FEZ and the other > 18 km north of the FEZ where humans were never evacuated, and radiation levels were at or near background levels. The mean ambient dose rate measured 1-m above ground at each camera site using a handheld dose-rate meter (Hitachi ALOKA TSC171) was 2.74 μSv/h in the FEZ (range: 0.30–8.35 μSv/h) and 0.19 μSv/h in the inhabited reference area (range: 0.09–0.47 μSv/h). Both areas were relatively rural with natural and plantation forests making up more than 75% of the land (the remainder was composed of < 10% rice paddy fields, < 10% other agricultural fields, and < 5% urban areas)^46^. The landscape was characterized by mountainous terrain with numerous rivers and streams. Valleys were often inhabited and developed for agricultural use such as rice paddies, while hilltops and slopes remained forested. Rice paddies and other agricultural areas were abandoned within the FEZ, but remained active in the inhabited area during the study period. To minimize any effects of elevation or land use on the composition of vertebrate scavengers between our two general study areas^48^, we limited our experimental sites to upland locations within the Abukuma Highlands. The mean elevation at our camera sites was 438 m (range: 49–754 m). The average temperature during the study period was approximately 23 °C (annual mean: 14 °C), and the annual precipitation for the region was 1093 mm in 2018^59^.

Based on a survey by Lyons et al.^48^, relatively abundant potential scavenger species in Fukushima and the surrounding region included wild boar, raccoon dog (*Nyctereutes procyonoides*), raccoon (*Procyon lotor*), masked palm civet (*Paguma larvata*), and red fox (*Vulpes vulpes*). Of these species, wild boar were 3–4 times more abundant in the FEZ than inhabited areas, and had higher relative abundance at upland sites (> 90 m) than raccoon dogs, raccoons, and civets. Although Asiatic black bears (*Ursus thibetanus*) are known dominant scavengers^31^, they were recorded rarely in the FEZ and thus were not expected to be important scavengers in this study^48^.

### Experimental design

All scavenging trials were conducted from May 28 to July 20, 2018, and were balanced across two carcass sizes (mice, *Mus musculus*; rabbits, *Sylvilagus* sp.), two forest cover types (deciduous broadleaf and evergreen conifer), and two zones with varying levels of human activity (FEZ, reference area). Carcass size is known to influence scavenger composition in some cases^22,53^, so we used two different carcass sizes to better capture the diversity of vertebrate scavengers in our study area: mice (mean ± SD = 12.0 ± 1.4 g) and rabbits (1395.7 ± 246.1 g). All carcasses were obtained frozen from Japanese pet food suppliers (Ryoshindo; Kasugai, Aichi, Japan; Tsukiyono farm: Minakami, Gunma, Japan). No “wild-type” (brown) carcasses were available in bulk, so all carcasses had white pelage. However, as many mammalian scavengers detect carcasses primarily via olfactory cues^60–62^ and because carcass color was consistent across all treatments, we did not expect it to significantly bias our results. Smaller carrion does not persist in the environment as long as larger carcasses and are more frequently usurped by invertebrates and microbes prior to arrival of vertebrate scavengers^22,53^, so we doubled the number of mouse trials to allow sufficient power for detecting differences in scavenger community and efficiency. We weighed all carcasses prior to use and calculated average weight for both carcass sizes.

**Figure 1:**
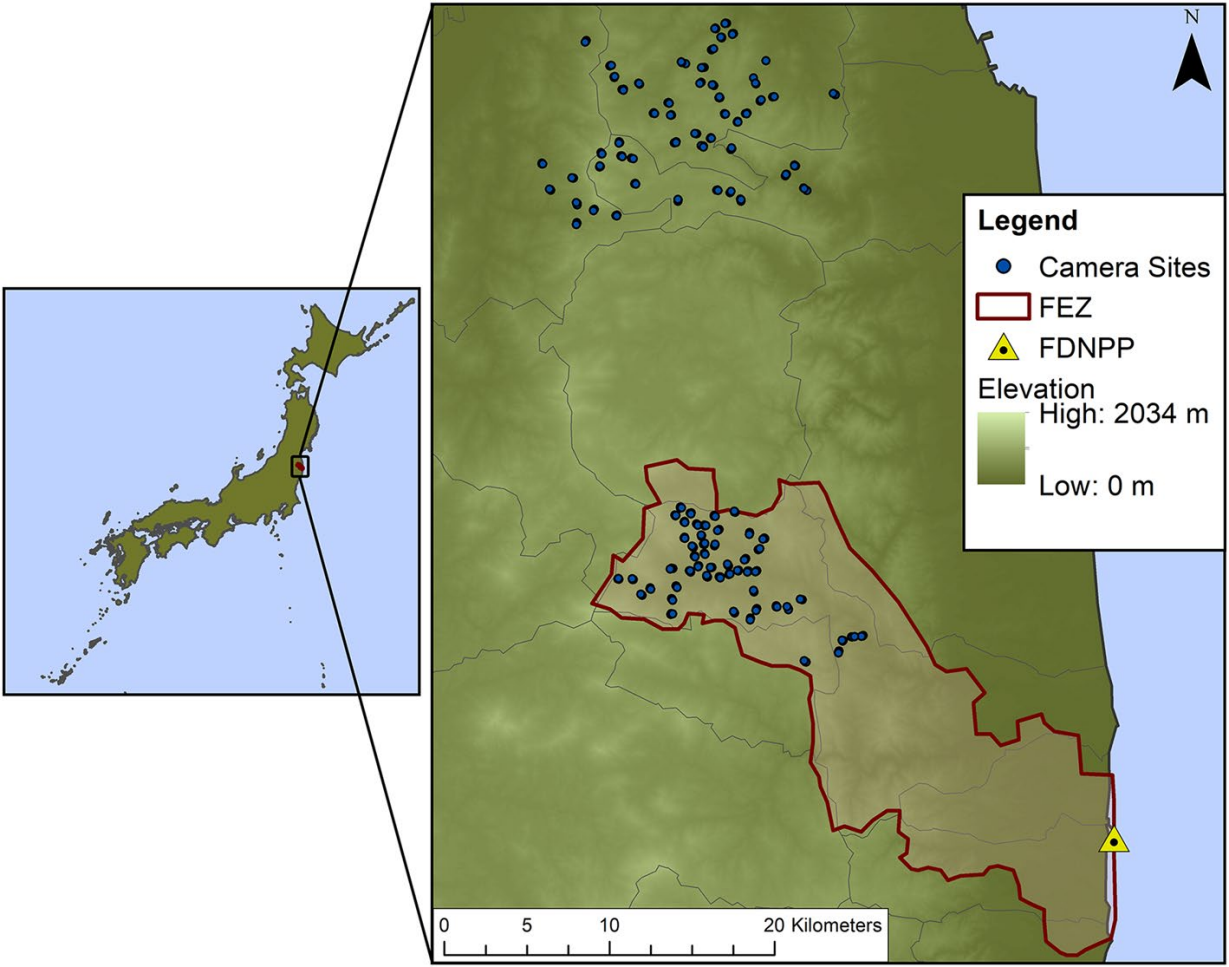
Map of camera sites used for scavenging trials in and around the Fukushima Exclusion Zone (FEZ) in relation to the Fukushima Daiichi Nuclear Power Plant (FDNPP). Inset map includes the location of the FEZ within Japan. This figure was created using ArcMap 10.8.1 (https://www.esri.com/en-us/arcgis/products/arcgis-desktop/resources).

We identified 100 unique sites (50 each in the FEZ and reference area) spaced at least 0.5 km apart (Fig. 1). We selected sites according to forest type (i.e., deciduous broadleaf and evergreen conifer forest), forest patch size (≥ ~ 0.70 ha), and locations where we had previously obtained landowner permission. We divided sites evenly between deciduous broadleaf (BL) and evergreen conifer (EC) forests using Geographic Information System (GIS) land cover data in ArcMap (v10.1) obtained from the Japan Aerospace Exploration Agency^63^. We conducted 300 scavenging trials (200 mice, 100 rabbit trials) for a total of 50 mouse and 25 rabbit trials per forest type per zone. Each site had three replicate trials (two mice, one rabbit carcass), and only one trial per site was active at any time during the study. After a preliminary screening of results during the field season, we conducted an additional 17 “redo” trials during this same period to account for suspected failed trials where the camera malfunctioned or the carcass was shifted out of the frame prior to depletion or removal, resulting in 317 total trials. To avoid habituating scavengers to trial locations within the same site, we spatially and temporally varied carcass placement for each trial. In addition to shifting subsequent carcasses at least 50 m from previous trial locations at the same site, we allowed a temporal buffer period after placing a carcass (7 days after mice; 14 days after rabbits) before starting a new trial. We also removed any carcass remains after each trial to prevent visual/olfactory cues from biasing subsequent trials. To limit edge effects, we kept trial locations within the same forest patch and at least 25 m from a forest edge or road whenever possible.

To record scavenging events, we placed carcasses in front of Reconyx no-glow, infrared HyperFire PC900 remote sensing cameras (RECONYX, Inc., Holmen, Wisconsin, USA) mounted to a tree roughly 1 m away. We cleared all vegetation around the carcass to allow identification of scavengers/carcass fate and avoid accidental camera trigger. We programmed cameras to take a burst of three photos one second apart upon triggering of the motion-sensor, with a refractory period of 1 min. To detect smaller or ectothermic scavengers not reliably captured by the motion-sensor, we also programmed cameras to take a time lapse photo every 5 min. We monitored mouse carcasses for 5–7 days and rabbit carcasses for 7 days, which was a sufficient period for most carcasses to be scavenged or to decompose. We kept all rabbit carcasses within the camera view by staking them to the ground with metal stakes and non-relaxing snares attached to a hind leg. Mouse carcasses were staked using either a snare placed around the midsection of the mouse or were tied directly to the stake using black sewing thread. We placed carcasses at varying times throughout the day to avoid time-related bias within trials^22,44^.

### Analyses

#### Image processing

For each carcass trial, we identified all species that visited or scavenged a carcass from remote camera images. A “visit” consisted of a scavenger or non-scavenger species that appeared in the frame but did not interact with the carcass. We defined a scavenging event as any time a vertebrate scavenger consumed or manipulated a carcass (i.e., touching or moving it), with the carcass displaced, diminished, or missing in subsequent photos. Trials where the carcass was suddenly removed with no visible scavenger were classified as “unknown”. We processed all photos using the CPW Photo Warehouse to create timestamped detections for each scavenging event^64^. After reviewing the photos and evaluating the intervals between return visits by the same scavenger species, we considered any consecutive photos of a species at the same location within 10 min of the previous photo to be a single detection.

#### Scavenging efficiency

We defined scavenger efficiency as a vertebrate scavenger’s ability to locate and consume a carcass. To distinguish between carcasses fully scavenged by vertebrates and those partially scavenged by vertebrates but where invertebrates removed the majority of the carcass, we quantified both (1) the proportion of trials in which the majority of the carcass was removed by a vertebrate scavenger, and (2) the proportion of trials scavenged by a vertebrate (overall scavenging rate, no matter how much of the carcass was consumed). We also recorded the time in hours until a carcass was first scavenged (“detection time”) and entirely scavenged or decomposed with the exception of bones/hair (“persistence time”). All statistical analyses were performed with R (v3.4.1).

To test the effects of carcass size, habitat, and zone of human activity on whether or not a carcass was scavenged, we used Generalized Linear Models (GLM) with a binomial error distribution and a logit link. We created models based on a priori hypotheses with combinations of our three main variables (carcass size, habitat, zone of human activity) and their interactions. We also ran a separate model using only data from mouse carcasses to test if attaching the carcass to the stake via snare or thread had a significant effect on whether a carcass was scavenged.

For analyses of detection time, we included failed trials where the carcass fate was unclear (See “Results”) as long as the carcass was detected before the trial failed (i.e., we counted trials where the carcass was partially scavenged before it was removed from the frame as “detected”). We used the R package “survival”^65^ to calculate the probability of a carcass being detected over time. We used a log rank test to calculate χ^2^ for observed and expected events for each time step to evaluate the effects of carcass size, habitat, and human activity zone on detection. To test the effect of rain on carcass detection by vertebrate scavengers, we determined the presence or absence of rain while the carcass was still present by examining the time lapse photos and included the categorical variable rain (yes/no) in our models.

To analyze carcass persistence time, we excluded trials that failed due to camera malfunction or the carcass being moved out of the camera frame prematurely before a carcass was fully scavenged or decomposed. When carcass remains (i.e., flesh in addition to hair/bones) were present at the end of the trial period, we assigned the entire length of the trial as the persistence time. Similar to the detection analyses, we used the “survival” package in R to calculate the probability of a carcass persisting through time, and cox proportional hazards models to test the effects of carcass size, habitat, human activity zone, and their interactions on persistence time. We also tested for the effects of rain on carcass persistence.

#### Scavenger species composition

We assessed species composition in three ways across each of our response variables (i.e., human activity zone, habitat, and carcass size): overall species richness, species richness per trial, and the percent occurrence of scavenger species. Overall species richness was a count of the species detected across trials in each combination of the variables human activity zone, habitat, and carcass size. For this analysis we counted overall species richness separately for species observed scavenging as well as all observed species (scavengers and non-scavengers) to characterize the scavenger and general wildlife community composition across our two study areas. We also tested the effects of zone, habitat, and carcass size (and their interactions) on scavenger species richness within each trial. To do so, we fitted Generalized Linear Mixed Effects Regression (GLMER) models from the package “lme4”^66^ with a Poisson distribution, including “site” as a random effect. To quantify the overall percent occurrence of each scavenger species, we calculated the proportion of trials a species scavenged divided by the total number of trials. We then calculated percent occurrence for scavenger species across each subgroup of variables (e.g., percent occurrence of scavengers for each carcass size in each zone). We recorded the proportion of trials visited but not scavenged by both scavenger and non-scavenger species to get a better understanding of the entire wildlife community. To help characterize each species’ role in the scavenging community and identify dominant scavengers, we also calculated the proportion of trials for which each species was the first scavenger and the proportion of carcasses each species fully removed or consumed.

## Results

### Carcass fate and community composition

A total of 73.1% of all carcasses were scavenged at least once by a vertebrate, of which 25.6% were partially scavenged and 47.6% were fully scavenged (> 50% removed by a vertebrate). Scavenging rates were similar between human habitation zones (FEZ: 73.2%; reference: 73.0%) and habitats (BL: 75.5%; CF: 70.8%), but varied strongly by carcass size. Rabbit carcasses were scavenged significantly more than mouse carcasses, with only 1% un-scavenged by vertebrates compared to 40% of mouse carcasses (Z = 4.10, p < 0.0001). Neither human habitation (Z = 0.04, p = 0.97) nor habitat (Z = − 0.91, p = 0.36) significantly affected carcass fate. The presence of a metal snare on mice carcasses also had no effect on whether it was scavenged compared to carcasses attached via thread (Z = − 0.14, p = 0.89).

In total, we documented ≥ 20 vertebrate species, 13 of which were observed scavenging (Table 1). Overall species richness was similar but slightly higher inside the FEZ compared to the reference area for all species (19 vs. 16) and scavenger species (13 vs. 11). Mammalian mesocarnivores and generalist omnivores (n = 9) dominated scavenging communities in both zones, although we also documented scavenging by avian (n = 2) and reptilian (n = 2) taxa. The number of scavenger species was similar between habitats and carcass sizes, but scavenger species richness per trial varied by carcass size. As expected, rabbit carcasses had significantly higher average scavenger richness per carcass than mouse carcasses, with a maximum of six species scavenging a single carcass (rabbit: 2.36 ± 1.16; mouse: 1.33 ± 0.58; Z = 5.71, p < 0.0001). Average scavenger species richness at each trial was not significantly different between human habitation zones (FEZ: 1.85 ± 1.08; reference area: 1.72 ± 0.96; Z = − 0.68; p = 0.50) or habitats (BL: 1.83 ± 1.02; CF: 1.74 ± 1.02; Z = − 0.48; p = 0.63).

**Table 1:** Proportion of all experimentally placed carcasses (n = 309) visited and scavenged by all species across both carcass sizes (mice, rabbits) in Fukushima, Japan from May to July 2018, including trials from both the Fukushima Exclusion Zone (FEZ) and inhabited reference area.

Common name	Species	Mouse carcasses				Rabbit carcasses			
		Visited (%)	n (209)	Scavenged (%)	n (209)	Visited (%)	n (100)	Scavenged (%)	n (100)
**Mammalian**									
Field mouse^a^	*Apodemus* sp.	80.9	169	40.2	84	81.0	81	16.0	16
Wild boar	*Sus scrofa*	20.1	42	10.0	21	31.0	31	45.0	45
Raccoon dog	*Nyctereutes procyonoides*	10.5	22	9.1	19	49.0	49	63.0	63
Masked palm civet	*Paguma lavarta*	11.5	24	5.7	12	46.0	46	51.0	51
Japanese red fox	*Vulpes vulpes*	1.4	3	0.5	1	7.0	7	15.0	15
Japanese marten	*Martes melampus*	15.3	32	1.0	2	21.0	21	4.0	4
Japanese shrew-mole	*Urotrichus talpoides*	7.7	16	1.0	2	3.0	3	0.0	0
Japanese badger	*Meles anakuma*	7.2	15	0.0	0	10.0	10	0.0	0
Raccoon	*Procyon lotor*	0.0	0	0.0	0	0.8	1	1.0	1
Domestic dog	*Canis lupus familiaris*	0.0	0	0.0	0	0.8	1	1.0	1
Domestic cat	*Felis catus*	2.4	5	0.0	0	3.0	3	0.0	0
Japanese weasel	*Mustela itatsi*	1.0	2	0.0	0	1.0	1	0.0	0
Japanese hare	*Lepus brachyurus*	16.7	35	0.0	0	8.0	8	0.0	0
Japanese squirrel	*Sciurus lis*	11.5	24	0.0	0	6.0	6	0.0	0
Japanese serow	*Capricornis crispus*	11.0	23	0.0	0	12.0	12	0.0	0
Japanese macaque	*Macaca fuscata*	8.1	17	0.0	0	2.0	2	0.0	0
**Avian**									
Crow^a^	*Corvus* sp.	1.4	3	1.9	4	4.0	4	21.0	21
Black kite	*Milvus migrans*	0.5	1	1.4	3	1.0	1	1.0	1
Pheasant^a^		11.5	24	0.0	0	4.0	4	0.0	0
Bird (other)^a^		5.7	12	0.0	0	9.0	9	0.0	0
**Reptile**									
Japanese rat snake	*Elaphe climacophora*	1.0	2	1.4	3	5.0	5	6.0	6
Burrowing rat snake	*Euprepiophis conspicillata*	1.4	3	0.5	1	1.0	1	1.0	1
Snake (other)^a^		2.9	7	0.0	0	4.0	4	0.0	0
**Unknown**		6.2	13	5.3	11	6.0	6	8.0	8

^a^Not identified to species.

**Figure 2:**
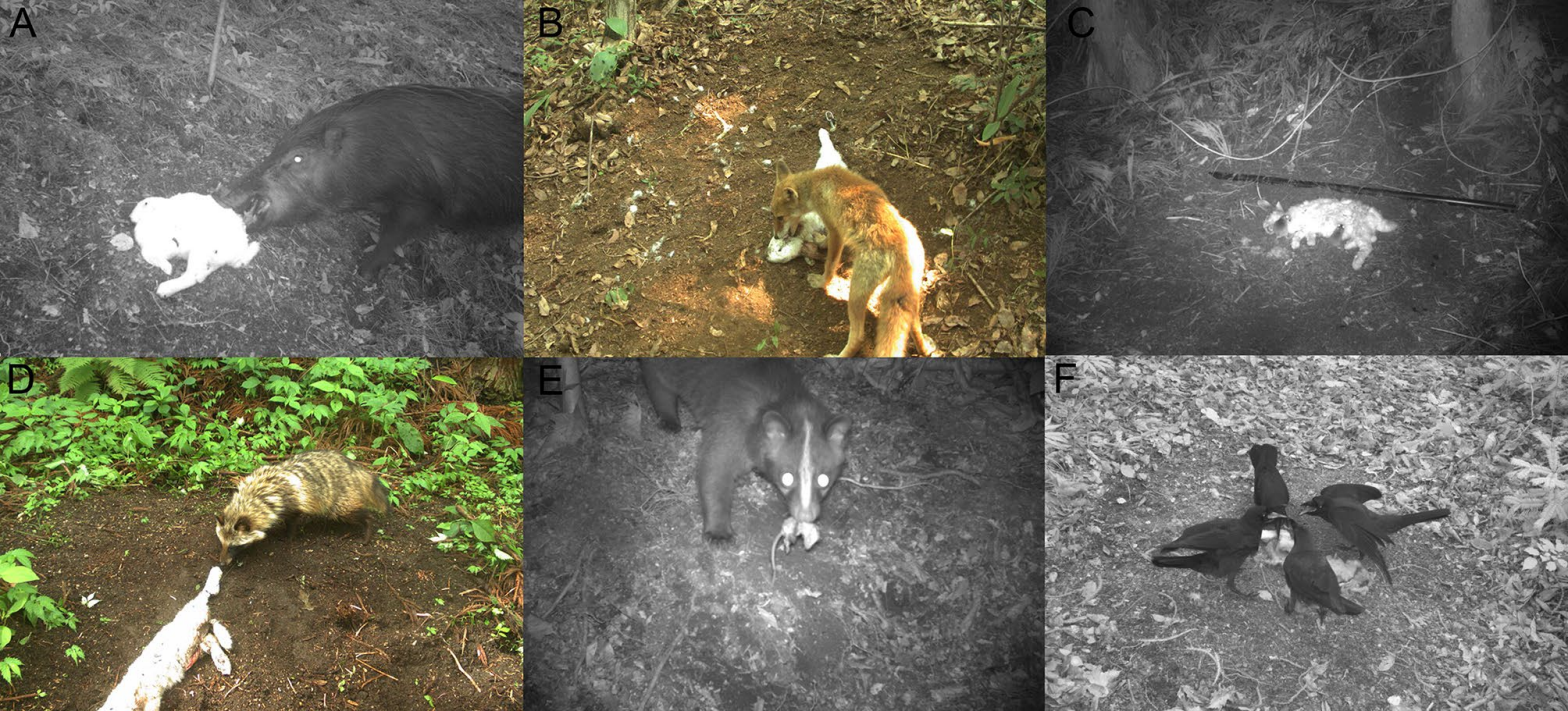
Frequent scavengers of rabbit and mouse carcasses in Fukushima, Japan captured by remote camera from May to July 2018. (**A**) wild boar (*Sus scrofa*), (**B**) red fox (*Vulpes vulpes*), (**C**) field mice (*Apodemus* sp.), (**D**) raccoon dog (*Nyctereutes procyonoides*), (**E**) masked palm civet (*Paguma larvata*), (**F**) crows (*Corvus* sp.).

**Figure 3:**
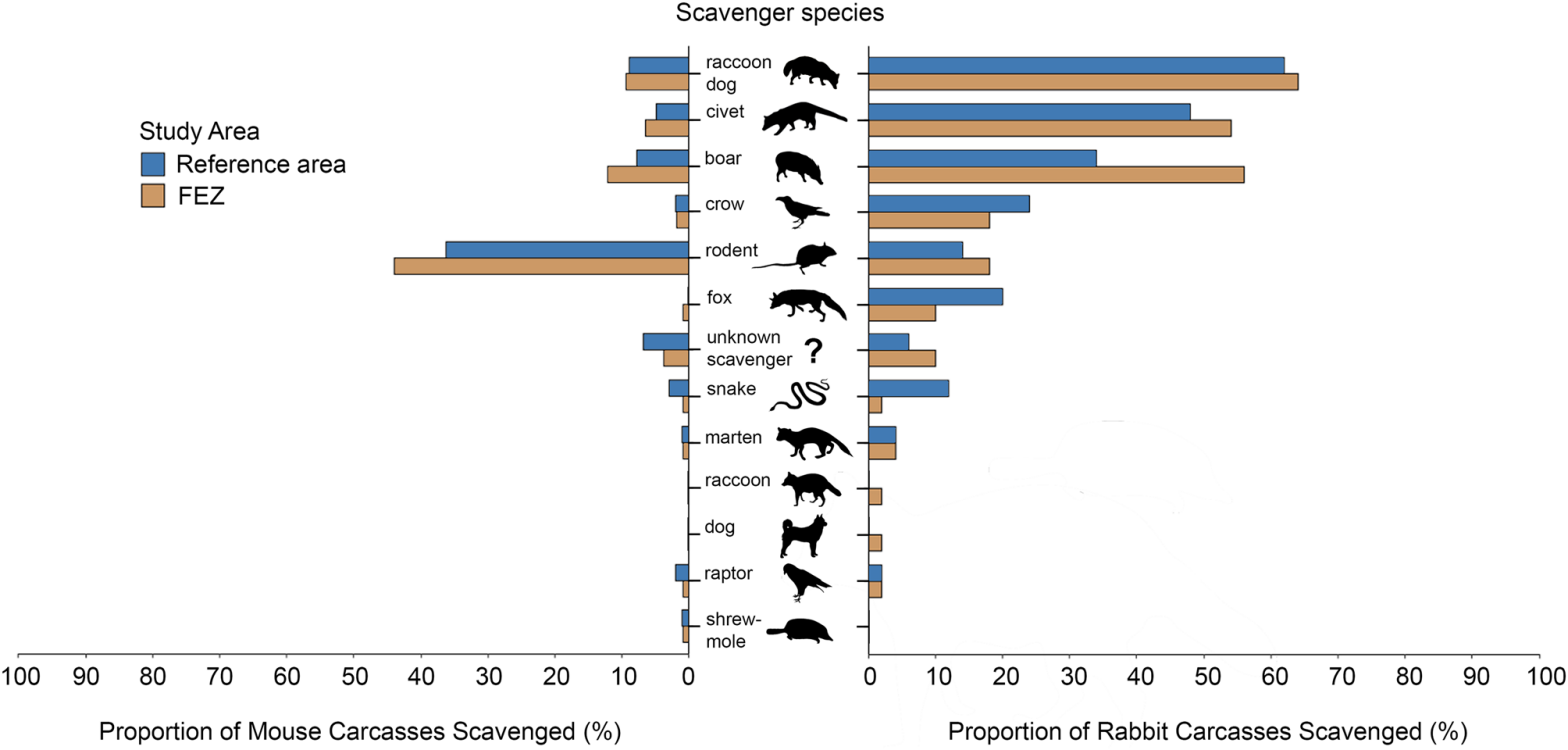
Proportion of experimentally placed mouse carcasses (n = 209) and rabbit carcasses (n = 100) partially or fully scavenged by different vertebrate scavengers in the Fukushima Exclusion Zone (FEZ) and inhabited reference area during May–July 2018.

Percent occurrence of scavenger species varied strongly by carcass size for most species (Table 1). The most frequent scavenger of mouse carcasses were rodents (40%), while the top scavengers of rabbit carcasses were raccoon dogs (63%), civets (51%), and wild boar (45%) (Fig. 2). Percent occurrence of scavenger species also differed across human habitation zones for several species (Fig. 3). Wild boar and rodents both scavenged at a higher proportion of all trials in the FEZ than reference area. Boar scavenged at 22% more rabbit trials in the FEZ, while foxes and snakes showed the opposite trend—both scavenged at 10% more rabbit trials in the reference area.

### Carcass detection

Of the trials where a carcass was detected by a vertebrate before the trial failed or ended (n = 225), the average detection time differed significantly between carcass sizes (χ^2^_1_ = 43.87, p < 0.0001), but not human habitation zones (χ^2^_1_ = 3.4, p = 0.07) or habitats (χ^2^_1_ = 0.35, p = 0.56). The mean detection time for mouse carcasses was 33 h quicker than rabbit carcasses (20 vs. 53 h). Scavengers also detected carcasses slightly faster without rain, but the effect was not significant (χ^2^_1_ = 3.32, p = 0.20). Detection times averaged 1.6 and 5 h faster for mouse and rabbit carcasses without rain, respectively.

Of the carcasses scavenged by vertebrates, rodents were the first scavenger at a high percentage of mouse carcasses (63.5%), followed by wild boar (12.0%), and then civet and raccoon dog (both 7.1%). For rabbit carcasses, the most common first scavengers were civet (24.2%), raccoon dog (22.2%), rodents (14.1%), and boar (12.1%). Given that rodents were common scavengers but unable to fully consume rabbit carcasses in our study, we also removed scavenging events by rodents and reanalyzed the data to investigate the most common first scavenger species other than rodents. From these analyses, the most common first scavengers of mouse carcasses were wild boar (25.8%), raccoon dog (24.2%), and civet (16.7%), whereas the first scavengers of rabbit carcasses were typically raccoon dog (30.0%), civet (27.8%), crow (13.5%), and wild boar (12.4%).

### Carcass persistence and removal

Carcass persistence varied by carcass size but not human habitation (χ^2^_1_ = 0.24, p = 0.62) or habitat (χ^2^_1_ = 0.47, p = 0.49). Rabbit carcasses persisted an average of 71.8 h longer than mouse carcasses (χ^2^_1_ = 25.2, p < 0.0001). Although more species were observed scavenging at rabbit carcasses than mouse carcasses (12 vs. 11 spp.), the smaller size of mouse carcasses enabled them to be fully removed by a greater diversity of scavengers than rabbit carcasses (10 vs. 6 spp.). For example, rodents clearly scavenged easily accessible parts of rabbit carcasses (e.g., face, ears, appendages, anus) (Fig. 2), and snakes were observed biting and coiling around rabbit carcasses, but neither were able to consume substantial amounts of the larger carcasses. Instead, raccoon dogs and wild boar were frequently the final scavengers and were strong enough to consistently remove the entire rabbit carcass from the snare.

**Figure 4:**
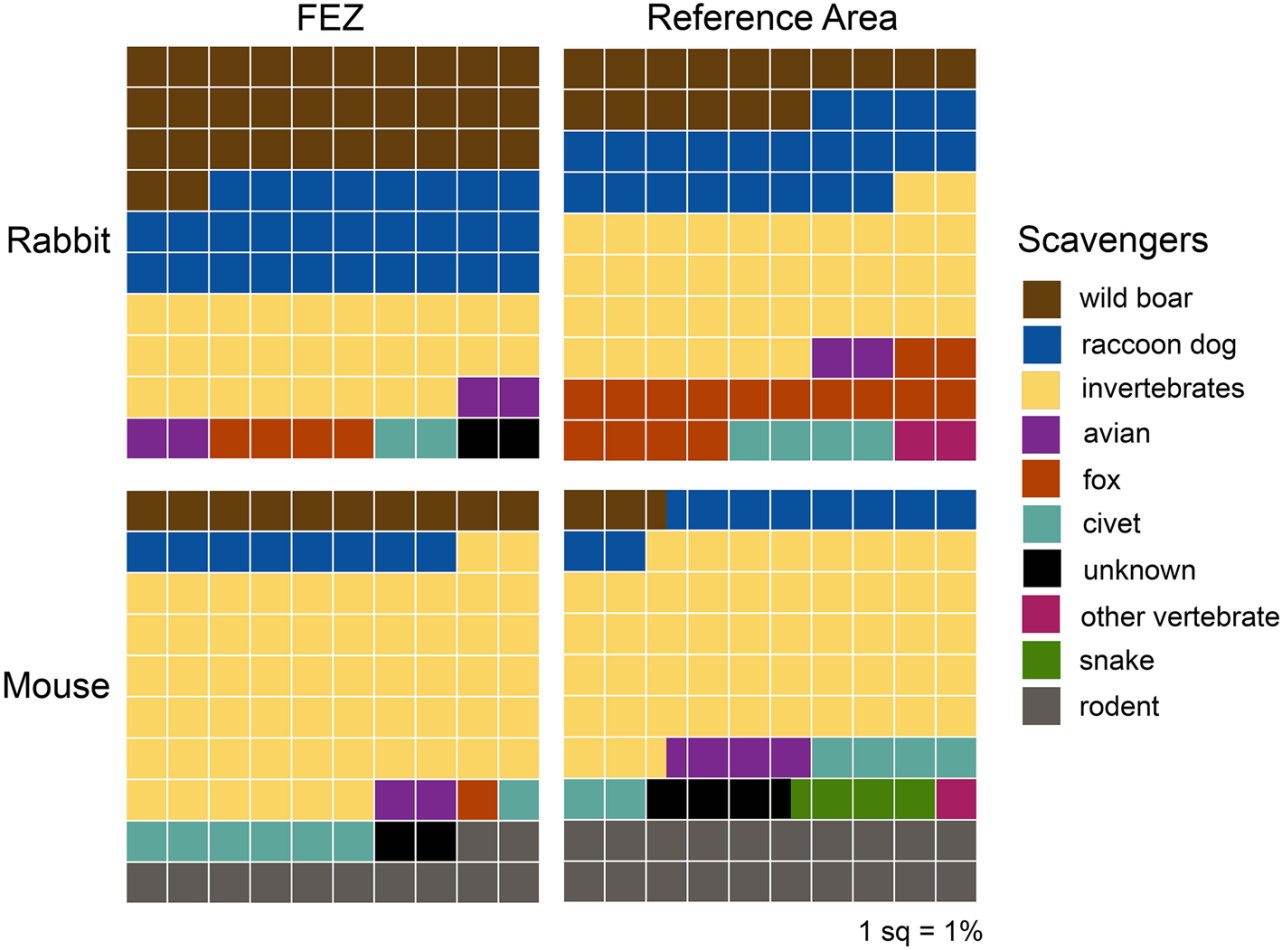
Experimentally placed mouse carcasses (n = 175) and rabbit carcasses (n = 100) removed by different scavenger species in both the Fukushima Exclusion Zone (FEZ) and inhabited reference area from May to July 2018. Scavengers were considered to have removed a carcass if they consumed or removed > 50% of the flesh.

**Figure 5:**
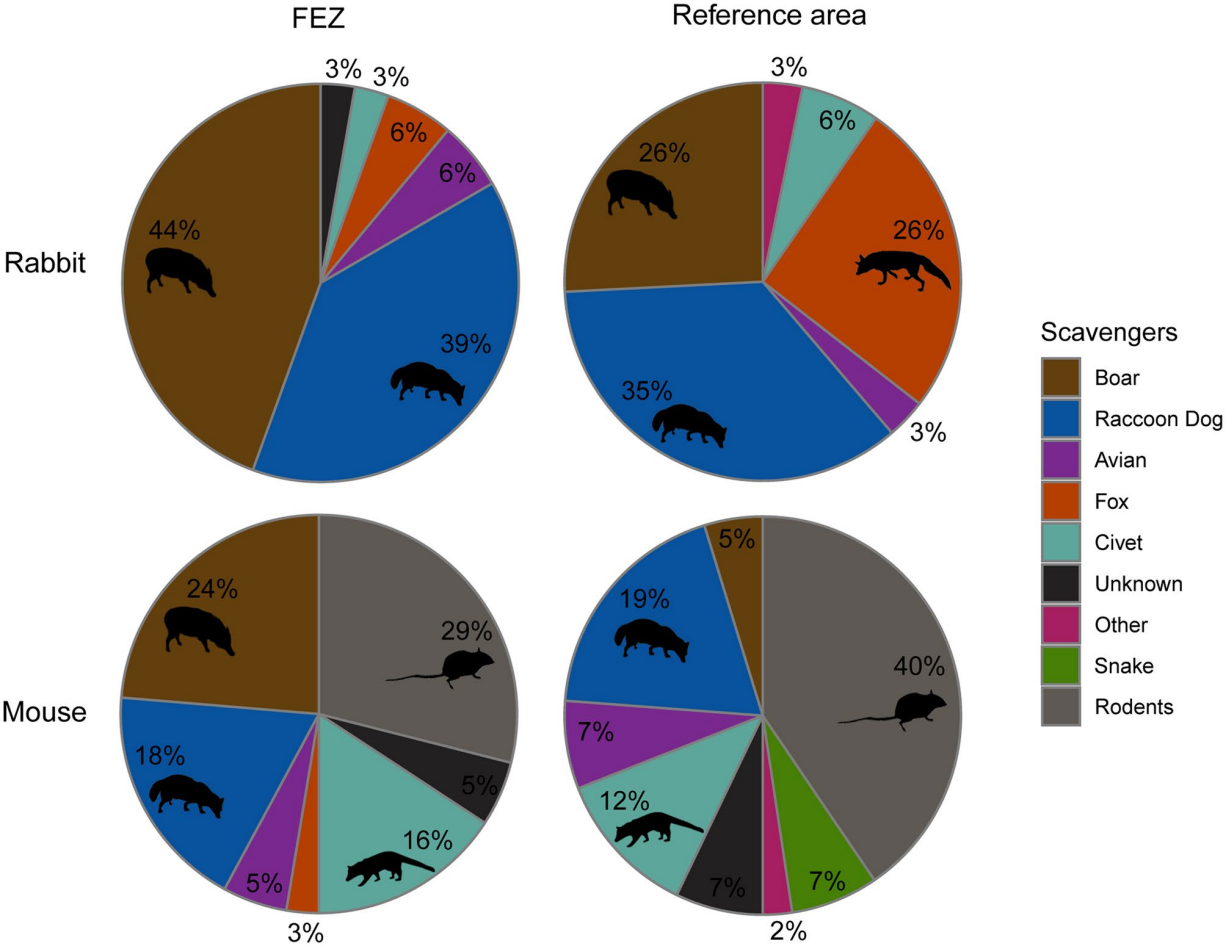
Proportion of vertebrate-removed carcasses scavenged by species for each carcass size in the Fukushima Exclusion Zone (FEZ) and inhabited Reference area.

Wild boar removed substantially more carcasses in FEZ compared to the reference area—a trend which was apparent across both carcass sizes (Fig. 4). Wild boar removed 32% of rabbit carcasses in the FEZ but only 16% in the reference area, where more rabbit carcasses were removed by foxes (16%) and invertebrates (38%). The relatively small size of carcasses in our study led to intense competition with invertebrate scavengers, which removed 30–40% of rabbit carcasses and 50–60% of all mouse carcasses in both the FEZ and reference area. However, of the carcasses removed by vertebrate scavengers, wild boar took five times more mice and 18% more rabbit carcasses in the FEZ than reference area (Fig. 5). The proportion of vertebrate-removed carcasses taken by raccoon dogs was roughly similar across the FEZ and reference area for both carcass sizes (mouse: 18 vs. 19%; rabbit: 39% vs. 36%), but other scavenger species removed more carcasses in the reference area where boar populations and carcass removal were lower. For example, rodents removed 29% of mouse carcasses taken by vertebrates in the FEZ compared to 40% in the reference area, and foxes removed 6% of rabbit carcasses in the FEZ and 26% in the reference area.

**Figure 6:**
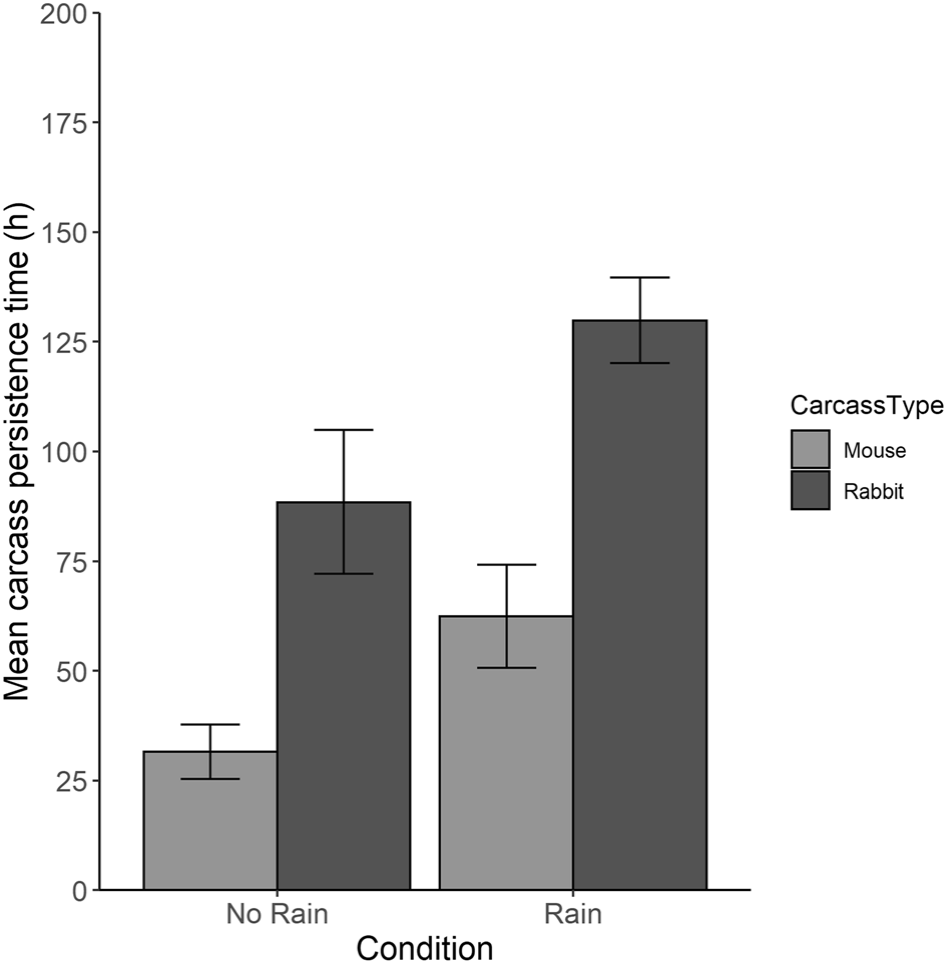
Mean time in hours (with 95% confidence intervals) until carcasses (n = 270) were fully scavenged or decomposed with and without rain during the experimental scavenging trials in the Fukushima Exclusion Zone (FEZ) and inhabited reference area.

Both carcass sizes lasted longer if it rained at least once while the carcass was present compared to trials without rain (χ^2^_1_ = 25.3, p < 0.0001) (Fig. 6). Mouse carcasses persisted twice as long when rain occurred during the trials (x̄ = 62.5 ± 51 h, n = 74) than during trials where no rain occurred (x̄ = 31.6 ± 30 h, n = 92). Similarly, rabbit carcasses persisted 1.5 times longer when rain occurred (x̄ = 129.8 ± 40.5 h, n = 69 vs. x̄ = 88.5 ± 42.9 h, n = 29). Invertebrate scavengers also removed a higher proportion of carcasses in trials where rain occurred (50 vs. 44%). When analyzed by carcass size, the effects of rain were greater for rabbit carcasses; invertebrates removed more than twice as many rabbit carcasses when it rained (39% vs. 17%).

## Discussion

Vertebrate scavengers were equally as diverse and efficient in the evacuated FEZ as they were in the surrounding inhabited landscape. Contrary to our expectations, overall scavenging rates were similarly high (~ 70%) across zones regardless of human presence or habitat. High scavenging rates indicate some ecosystem services such as carrion removal have not been negatively impacted by the 2011 nuclear accident. The increased abundance of wild boar in the FEZ following human evacuation^48,67^ did not increase the total proportion of vertebrate-scavenged carcasses, but instead shifted the allocation of carrion resources among facultative scavengers. As predicted, carcass size influenced most aspects of scavenging dynamics, including the detection and persistence time of carcasses as well as scavenger identity and species richness. Mammalian mesocarnivores and generalist omnivores dominated the vertebrate scavenger community, making up 69% of scavenger species and removing more than half of all carcasses. Although ecosystems lacking obligate scavengers or large predators may be more vulnerable to fluctuations in the abundance of common scavengers^26^, our results suggest a diversity of facultative scavengers and efficient invertebrates contribute to the functional redundancy of small carrion removal in Fukushima. By characterizing the scavenging community in the FEZ, we also provide potential pathways of nutrient and radiocesium redistribution in a radiologically contaminated ecosystem. Given the diversity of facultative scavengers we observed and the tendency for some frequent scavengers like boar and rodents to accumulate high (albeit variable) levels of radiocesium^68,69^, the implications of scavenging behavior on radiocesium dynamics may warrant future research, as scavenging could lead to radiocesium circulating at higher trophic levels.

Overall, carcass size affected scavenging dynamics more than habitat or human activity zone. As we hypothesized, rabbit carcasses persisted significantly longer (~ 2.5× longer) and were more likely to be scavenged by a greater number of vertebrate species than the smaller mouse carcasses^22,70,71^. Mesocarnivores spent more time at rabbit carcasses, and we recorded several instances of inter- and intra-specific interactions between individuals fighting for dominance of carrion resources. Carcass size strongly influenced the scavengers’ ability to dominate carrion resources, as smaller scavengers (i.e., rodents, snakes) were able to physically remove mouse but not rabbit carcasses. For example, civets transitioned from being one of the top scavengers of mice^72^ and the second-most frequent scavengers at rabbit carcasses (this study), to being the least common scavenger at deer carcasses^31^. This shift implies a threshold in carcass size exists where size disparity prevents accessibility and/or risks attracting more dominant competitors. In contrast, both the occurrence of and carcass removal by bigger scavengers (e.g., wild boar, raccoon dog, and fox) generally seem to increase as carcass sizes increase^31,72–74^. Despite vertebrate scavengers detecting mouse carcasses faster due to their rapid decomposition and release of olfactory cues^20,75^, the mice’s smaller size also enables removal by invertebrates at higher rates^76^. For example, in our study carrion beetles frequently buried mouse carcasses within hours of placement, thus monopolizing the carcasses as vertebrates rarely located buried carcasses. The high rate of invertebrate scavenging indicates intense competition with vertebrate scavengers for carrion resources that increases the functional redundancy of carcass removal services^2,77^, but may also have implications for radiocesium redistribution among vertebrate and invertebrate scavengers.

Although larger boar populations in the FEZ corresponded to higher occurrences of wild boar at carcasses in addition to boar removing more carcasses, the overall proportion of carcasses scavenged by vertebrates was similar between zones. The flexible diets of facultative scavengers dominating our scavenging community might have reduced competition for carrion resources among vertebrates during warm months^69,78–80^, leading to a shift in resource allocation rather than an increase in the overall number of carcasses scavenged. Both foxes and rodents removed substantial proportions of carcasses in the reference area but less in the FEZ, where boar scavenged more carcasses (Figs. 4, 5). Surprisingly, despite rodents removing more carcasses in the reference area, they occurred at more carcasses and scavenged at higher rates in the FEZ (Fig. 3). We suspected higher densities of rodents increased their probability of encountering mouse carcasses compared to boar^55^, but boars’ larger size allowed for quicker consumption of carcasses. However, the mean persistence time for mouse carcasses removed by rodents was more than three times faster than those taken by boar. Thus, it remains unclear why rodents scavenged more but removed less mouse carcasses in the FEZ. It’s possible the discrepancy in rodent occurrence between evacuated and inhabited zones may be related to other factors such as land use changes after evacuation (e.g., abandonment of farms; succession of rice paddies to grassland)^81,82^ and resulting effects on rodent populations, but no research on the subject has been published thus far. As large social mammals, wild boar can dominate food resources and change other animals’ feeding behavior, including that of large carnivores^83–85^. In the absence of larger predators like bear or wolves (wolves have been extinct in Japan since the early 1900s)^31^, boar have the greatest mass of any scavenger in our study area. In Japan, when boar are present, raccoon dogs feed at different times and on steeper slopes to avoid competition over fallen fruit^84^. Unlike fruit, carrion is often a spatially unpredictable resource^86,87^ and raccoon dogs as mesocarnivores may be better adapted to rapidly detecting carrion than boar—however, mean detection times for raccoon dogs in this study were only slightly (but not significantly) faster than those of boar. Nonetheless, raccoon dogs occurred at more carcasses than boar and removed similar proportions of vertebrate-scavenged carcasses across zones, indicating their importance as scavengers even in areas with increased boar abundance.

Previous research in Japan has recorded high scavenging rates by raccoon dogs^72,77^, including studies where multiple larger competing scavengers (i.e., bear and boar) are present^31^ and functionally absent^73^, suggesting raccoon dogs are reliant on carrion across their range and throughout the year. Comparison across studies is complicated by differences in methodology and carcass sizes, but the percent occurrence of boar at mouse carcasses in this study (8–12%) was roughly similar to previous studies using mice^72^, while boar occurrence at rabbit carcasses (34–56%) was comparable to studies using deer carcasses^31^. Although the scavenging frequencies of boar and raccoon dog remained similar throughout seasons, Inagaki et al.^31^ observed higher scavenging by marten and fox in autumn, which indicates the relatively low occurrence of marten (2%) and fox (5%) in our study may be a seasonal effect. Additionally, the most frequent winter scavengers in northern Japan at sites without boar were raccoon dogs, martens, and foxes^73^. Facultative scavengers compete more strongly for carrion in the winter due to the scarcity of other food resources^53,56,73^, which may exacerbate the impact of wild boar dominating carcass removal in areas of high boar density. However, competition could also be somewhat alleviated by decreased invertebrate activity in colder months, resulting in longer carcass persistence and thus increased availability to vertebrate scavengers^53,73^.

We predicted habitat would impact scavenger species richness or efficiency of carcass removal given its documented effect on scavenging dynamics^36,53,56,76^, but we did not observe differences between deciduous broadleaf forests and evergreen conifer forests. Sugiura et al.^72^ observed similar carcass removal rates between deciduous forest and conifer plantations in Japan, indicating the two forest types supported similar scavenging communities. The effects of habitat on scavenging communities are complex and moderated by interactions with carcass size^53^ and spatial scale, as both landscape and local habitat characteristics influence scavenging communities^36,39^. We did not account for microhabitat or vegetation structure, but differences between the two forest types may not have been enough to affect the accessibility of carcasses by mammalian scavengers which mainly use olfactory cues to detect carcasses^60,61,88^. Therefore, our placement of carcasses in forest interiors likely contributed to this study’s relatively low scavenging rates (2.5%) by avian scavengers^31,73^ as many avian scavengers rely primarily on vision^61^. The larger size and longer persistence time of rabbit carcasses may have enabled crows to locate them on forest floors easier than mouse carcasses, resulting in higher proportions of rabbits scavenged by crows in this study (21% vs. 2%).

Our data suggest the functional role of rodents as scavengers may be understated in the literature. Rodents were the first vertebrates to scavenge carcasses at a surprisingly large proportion of trials (42%), more so than any other vertebrate. Several studies have observed scavenging by rodents using remote cameras^55,88–91^ but fewer have documented rodents as the most frequent or dominant scavenger^51^. Reliable detection of rodents may be problematic due to their small size and the variation in camera quality/set-up between studies. Our use of 5-min time lapse photos substantially increased our ability to detect scavenging by rodents even when they did not trigger the camera’s motion sensor—including multiple mice simultaneously feeding at the same rabbit carcass (Fig. 2). The time lapse settings also captured several instances of scavenging behavior (n = 11) by ectotherms that otherwise would have gone undetected by motion-triggered cameras. For example, some snakes attempted to scavenge rabbit carcasses, frequently biting and coiling around the rabbits’ faces despite the carcass being too large for the snakes to consume. Our detection of snakes via only time lapse photos helps illustrate why snakes have traditionally been underestimated as scavengers^92^, despite the widespread prevalence of scavenging behavior across snake species^62,86,93^.

Interestingly, the occurrence of rain during trials increased the persistence time of carcasses considerably regardless of carcass size. Although the effects of rain on carcass persistence are not well-studied, some studies have suggested precipitation may prolong carcass decomposition by decreasing vertebrate and invertebrate activity, or interfering with olfactory cues that lead to carcass detection by vertebrate scavengers^55,56,94^. The proportion of carcasses removed by invertebrates in this study was greater during trials with rain than without rain, suggesting decreased invertebrate activity was not responsible for the difference in persistence times. Detection by vertebrate scavengers was slightly but not significantly reduced by the occurrence of rain in this study, possibly because of other factors that impact detection like windspeed, humidity, and the amount/duration of rainfall, which were not measured in this study^54^. Additionally, rain is closely linked to temperature, which influences scavenging efficiency and carcass persistence^28,53,60^. Future research to clarify the interaction between temperature and precipitation, and how it influences invertebrate activity and detection by vertebrate scavengers, would increase our understanding of carcass persistence across seasons and climates.

## Conclusions

In this study, we examined the composition and efficiency of the vertebrate scavenger community in the evacuated FEZ and nearby areas unaffected by the 2011 accident. Our results indicate that despite the presence of radiological contamination and the increased boar abundance following human evacuation, vertebrate scavengers inside the FEZ are equally as diverse and efficient at assimilating carrion as in the surrounding landscape. Using time-lapse photography in addition to the camera’s motion-sensor allowed for the detection of a greater diversity of species often missed in scavenging studies, including extensive scavenging by mice and several instances of scavenging behavior by snakes. Overall, carcass size had a greater effect on scavenging dynamics than habitat or human activity through its role in carcass detection and persistence, as well as carcass use by vertebrate and invertebrate scavengers. Higher boar abundances in the FEZ did not increase scavenging rates but shifted the allocation of carrion resources among scavengers, with boar dominating more carcasses in the FEZ. Our results support recent work suggesting that in systems without obligate scavengers and large carnivores, changes in the abundance of common or dominant scavengers can influence scavenging dynamics. This work also adds to growing evidence that scavenging research should account for the effects of carcass size when identifying scavenger communities. Finally, by characterizing the scavenging community in a radiologically contaminated ecosystem, we illustrate potential pathways of radiocesium dynamics in the food web that previously may not have been considered.

## References

[1] Lim N, Kelt DA, Lim KK, Bernard H (2020). Vertebrate scavengers control abundance of diarrheal-causing bacteria in tropical plantations. Zool. Stud..

[2] Beasley, J. C., Olson, Z. H. & DeVault, T. L. Ecological role of vertebrate scavengers. In: *Carrion Ecology, Evolution and their Applications*. (eds Benbow, E.M., Tomberlin, J. & Tarone, A.) 107–127 (CRC Press, 2015).

[3] Ogada DL, Keesing F, Virani MZ (2012). Dropping dead: Causes and consequences of vulture population declines worldwide. Ann. N. Y. Acad. Sci..

[4] Reid WV (2005). Ecosystems and Human Well-Being-Synthesis: A Report of the Millennium Ecosystem Assessment.

[5] Wilson EE, Wolkovich EM (2011). Scavenging: How carnivores and carrion structure communities. Trends Ecol. Evol..

[6] Moleón M, Sánchez-Zapata JA, Selva N, Donázar JA, Owen-Smith N (2014). Inter-specific interactions linking predation and scavenging in terrestrial vertebrate assemblages. Biol. Rev..

[7] Fonseca CR, Ganade G (2001). Species functional redundancy, random extinctions and the stability of ecosystems. J. Ecol..

[8] Mori AS, Furukawa T, Sasaki T (2013). Response diversity determines the resilience of ecosystems to environmental change. Biol. Rev..

[9] Huijbers CM (2015). Limited functional redundancy in vertebrate scavenger guilds fails to compensate for the loss of raptors from urbanized sandy beaches. Divers. Distrib..

[10] Ceballos G (2015). Accelerated modern human–induced species losses: Entering the sixth mass extinction. Sci. Adv..

[11] Buechley ER, Şekercioğlu ÇH (2016). The Avian scavenger crisis: Looming extinctions, trophic cascades, and loss of critical ecosystem functions. Biol. Cons..

[12] Hill JE, DeVault TL, Wang G, Belant JL (2019). Anthropogenic mortality in mammals increases with the human footprint. Front. Ecol. Environ..

[13] Sebastián-González E (2019). Scavenging in the Anthropocene: Human impact drives vertebrate scavenger species richness at a global scale. Glob. Change Biol..

[14] Sebastián-González E (2020). Network structure of vertebrate scavenger assemblages at the global scale: Drivers and ecosystem functioning implications. Ecography.

[15] Marneweck CJ, Katzner TE, Jachowski DS (2021). Predicted climate-induced reductions in scavenging in eastern North America. Glob. Change Biol..

[16] Mokany K, Ash J, Roxburgh S (2008). Functional identity is more important than diversity in influencing ecosystem processes in a temperate native grassland. J. Ecol..

[17] Gagic V (2015). Functional identity and diversity of animals predict ecosystem functioning better than species-based indices. Proc. R. Soc. B Biol. Sci..

[18] Mateo-Tomás P, Olea PP, Selva N, Sánchez-Zapata JA (2019). Species and individual replacements contribute more than nestedness to shape vertebrate scavenger metacommunities. Ecography.

[19] Sebastián-González E (2021). Functional traits driving species role in the structure of terrestrial vertebrate scavenger networks. Ecology.

[20] DeVault TL, Rhodes OE, Shivik JA (2003). Scavenging by vertebrates: Behavioral, ecological, and evolutionary perspectives on an important energy transfer pathway in terrestrial ecosystems. Oikos.

[21] Allen ML, Elbroch LM, Wilmers CC, Wittmer HU (2015). The comparative effects of large carnivores on the acquisition of carrion by scavengers. Am. Nat..

[22] Moleón M, Sánchez-Zapata JA, Sebastián-González E, Owen-Smith N (2015). Carcass size shapes the structure and functioning of an African scavenging assemblage. Oikos.

[23] Gutiérrez-Cánovas C (2020). Large home range scavengers support higher rates of carcass removal. Funct. Ecol..

[24] Walker MA (2021). Factors influencing scavenger guilds and scavenging efficiency in Southwestern Montana. Sci. Rep..

[25] Winfree R, Fox J, Williams NM, Reilly JR, Cariveau DP (2015). Abundance of common species, not species richness, drives delivery of a real-world ecosystem service. Ecol. Lett..

[26] Mateo-Tomás P, Olea PP, Moleón M, Selva N, Sánchez-Zapata JA (2017). Both rare and common species support ecosystem services in scavenger communities. Glob. Ecol. Biogeogr..

[27] Butler JRA, du Toit JT (2002). Diet of free-ranging domestic dogs (*Canis familiaris*) in rural Zimbabwe: Implications for wild scavengers on the periphery of wildlife reserves. Anim. Conserv..

[28] DeVault TL, Olson ZH, Beasley JC, Rhodes OE (2011). Mesopredators dominate competition for carrion in an agricultural landscape. Basic Appl. Ecol..

[29] Ogada DL, Torchin ME, Kinnaird MF, Ezenwa VO (2012). Effects of vulture declines on facultative scavengers and potential implications for mammalian disease transmission. Conserv. Biol..

[30] Morales-Reyes Z (2017). Scavenging efficiency and red fox abundance in Mediterranean mountains with and without vultures. Acta Oecol..

[31] Inagaki A (2020). Vertebrate scavenger guild composition and utilization of carrion in an East Asian temperate forest. Ecol. Evol..

[32] Blazquez M, Sanchez-Zapata JA, Botella F, Carrete M, Eguía S (2009). Spatio-temporal segregation of facultative avian scavengers at ungulate carcasses. Acta Oecol..

[33] Inger R, Cox DTC, Per E, Norton BA, Gaston KJ (2016). Ecological role of vertebrate scavengers in urban ecosystems in the UK. Ecol. Evol..

[34] Hill JE, DeVault TL, Beasley JC, Rhodes OE, Belant JL (2018). Effects of vulture exclusion on carrion consumption by facultative scavengers. Ecol. Evol..

[35] Olson Z, Beasley J, DeVault TL, Rhodes OE (2012). Scavenger community response to the removal of a dominant scavenger. Oikos.

[36] Pardo-Barquín E, Mateo-Tomás P, Olea PP (2019). Habitat characteristics from local to landscape scales combine to shape vertebrate scavenging communities. Basic Appl. Ecol..

[37] Turner KL, Conner LM, Beasley JC (2020). Effect of mammalian mesopredator exclusion on vertebrate scavenging communities. Sci. Rep..

[38] Ohashi H (2013). Differences in the activity pattern of the wild boar *Sus scrofa* related to human disturbance. Eur. J. Wildl. Res..

[39] Saito M, Koike F (2013). Distribution of wild mammal assemblages along an urban–rural–forest landscape gradient in warm-temperate East Asia. PLoS ONE.

[40] Gaynor KM, Hojnowski CE, Carter NH, Brashares JS (2018). The influence of human disturbance on wildlife nocturnality. Science.

[41] Tsunoda M (2019). Human disturbance affects latrine-use patterns of raccoon dogs. J. Wildl. Manag..

[42] Watabe R, Saito MU (2021). Effects of vehicle-passing frequency on forest roads on the activity patterns of carnivores. Landsc. Ecol. Eng..

[43] Luna Á, Romero-Vidal P, Arrondo E (2021). Predation and scavenging in the city: A review of spatio-temporal trends in research. Diversity.

[44] Huijbers CM, Schlacher TA, Schoeman DS, Weston MA, Connolly RM (2013). Urbanisation alters processing of marine carrion on sandy beaches. Landsc. Urban Plan..

[45] Fukushima Prefectural Government. *Transition of evacuation designated zones*. https://www.pref.fukushima.lg.jp/site/portal-english/en03-08.html. (2019). Accessed 20 Apr 2022.

[46] Steinhauser G, Brandl A, Johnson TE (2014). Comparison of the Chernobyl and Fukushima nuclear accidents: A review of the environmental impacts. Sci. Total Environ..

[47] Center for International Earth Science Information Network (CIESIN)—Columbia University. (NASA Socioeconomic Data and Applications Center (SEDAC), Palisades, NY, 2018).

[48] Lyons PC, Okuda K, Hamilton MJ, Hinton TG, Beasley JC (2020). Rewilding of Fukushima’s human evacuation zone in the presence of radioactive stressors. Front. Ecol. Environ..

[49] Deryabina TG (2015). Long-term census data reveal abundant wildlife populations at Chernobyl. Curr. Biol..

[50] Webster SC (2016). Where the wild things are: Influence of radiation on the distribution of four mammalian species within the Chernobyl Exclusion Zone. Front. Ecol. Environ..

[51] Schlichting PE, Love CN, Webster SC, Beasley JC (2019). Efficiency and composition of vertebrate scavengers at the land–water interface in the Chernobyl Exclusion Zone. Food Webs.

[52] Newsome TM (2021). Monitoring the dead as an ecosystem indicator. Ecol. Evol..

[53] Turner KL, Abernethy EF, Mike Conner L, Rhodes OE, Beasley JC (2017). Abiotic and biotic factors modulate carrion fate and vertebrate scavenging communities. Ecology.

[54] Ruzicka RE, Conover MR (2012). Does weather or site characteristics influence the ability of scavengers to locate food?. Ethology.

[55] Paula JJS (2015). Camera-trapping as a methodology to assess the persistence of wildlife carcasses resulting from collisions with human-made structures. Wildl. Res..

[56] Selva N, Jędrzejewska B, Jędrzejewski W, Wajrak A (2005). Factors affecting carcass use by a guild of scavengers in European temperate woodland. Can. J. Zool..

[57] Nakama S, Yoshimura K, Fujiwara K, Ishikawa H, Iijima K (2019). Temporal decrease in air dose rate in the sub-urban area affected by the Fukushima Dai-ichi Nuclear Power Plant accident during four years after decontamination works. J. Environ. Radioact..

[58] Ministry of the Environment of Japan. *Off-Site Environmental Remediation in Affected Areas in Japan*. http://josen.env.go.jp/en/decontamination/ (2020). Accessed 20 Apr 2022.

[59] Japan Meteorological Agency. *Climate in Namie in 2018: Monthly Overview Data*. http://www.data.jma.go.jp/obd/stats/etrn/view/monthly_a1.php?prec_no=36&block_no=0295&year=2018&month=7&day=&view=p1 (2018). Accessed 1 Apr 2019.

[60] De Vault TL, Brisbin J, Lehr I, Rhodes J, Olin E (2004). Factors influencing the acquisition of rodent carrion by vertebrate scavengers and decomposers. Can. J. Zool..

[61] Kane A, Healy K, Guillerme T, Ruxton GD, Jackson AL (2017). A recipe for scavenging in vertebrates—The natural history of a behaviour. Ecography.

[62] Natusch DJD, Lyons JA, Shine R (2017). How do predators and scavengers locate resource hotspots within a tropical forest?. Aust. Ecol..

[63] Japan Aerospace Exploration Agency. *High-resolution land use land cover map of Japan (ver.16.09)*. https://www.eorc.jaxa.jp/ALOS/en/lulc/lulc_index.htm (2011). Accessed 1 Apr 2019.

[64] Newkirk, E. S. *CPW Photo Warehouse*. http://cpw.state.co.us/learn/Pages/ResearchMammalsSoftware.aspx (2016). Accessed 1 Apr 2019.

[65] Therneau, T. M. A Package for Survival Analysis in R. *R package version 3.3-1* (2022).

[66] Bates D, Mächler M, Bolker B, Walker S (2015). Fitting linear mixed-effects models using lme4. J. Stat. Softw..

[67] Anderson D (2021). Introgression dynamics from invasive pigs into wild boar following the March 2011 natural and anthropogenic disasters at Fukushima. Proc. R. Soc. B Biol. Sci..

[68] Ishiniwa H, Onuma M, Tamaoki M (2022). Behavior of Radionuclides in the Environment III.

[69] Nemoto Y (2020). Effects of ^137^Cs contamination after the TEPCO Fukushima Dai-ichi Nuclear Power Station accident on food and habitat of wild boar in Fukushima Prefecture. J. Environ. Radioact..

[70] Olson ZH, Beasley JC, Rhodes OE (2016). Carcass type affects local scavenger guilds more than habitat connectivity. PLoS ONE.

[71] DeVault TL, Seamans TW, Linnell KE, Sparks DW, Beasley JC (2017). Scavenger removal of bird carcasses at simulated wind turbines: Does carcass type matter?. Ecosphere..

[72] Sugiura S, Tanaka R, Taki H, Kanzaki N (2013). Differential responses of scavenging arthropods and vertebrates to forest loss maintain ecosystem function in a heterogeneous landscape. Biol. Cons..

[73] Enari H, Enari HS (2021). Not avian but mammalian scavengers efficiently consume carcasses under heavy snowfall conditions: A case from northern Japan. Mamm. Biol..

[74] Selva N, Jedrzejewska B, Jedrzejewski W, Wajrak A (2003). Scavenging on European bison carcasses in Bialowieza primeval forest (eastern Poland). Ecoscience.

[75] Jojola-Elverum SM, Shivik JA, Clark L (2001). Importance of bacterial decomposition and carrion substrate to foraging brown treesnakes. J. Chem. Ecol..

[76] Abernethy EF, Turner KL, Beasley JC, Rhodes OE (2017). Scavenging along an ecological interface: Utilization of amphibian and reptile carcasses around isolated wetlands. Ecosphere.

[77] Sugiura S, Hayashi M (2018). Functional compensation by insular scavengers: The relative contributions of vertebrates and invertebrates vary among islands. Ecography.

[78] Matsuo R, Ochiai K (2009). Dietary overlap among two introduced and one native sympatric carnivore species, the raccoon, the masked palm civet, and the raccoon dog, in Chiba Prefecture, Japan. Mammal Study.

[79] Drygala F, Zoller H (2014). Diet composition of the invasive raccoon dog (*Nyctereutes procyonoides*) and the native red fox (*Vulpes vulpes*) in north-east Germany. Hystrix Italian J. Mammal..

[80] Elmeros M (2018). The diet of feral raccoon dog (*Nyctereutes procyonoides*) and native badger (*Meles meles*) and red fox (*Vulpes vulpes*) in Denmark. Mammal Res..

[81] Sekizawa R, Ichii K, Kondo M (2015). Satellite-based detection of evacuation-induced land cover changes following the Fukushima Daiichi nuclear disaster. Remote Sensing Lett..

[82] Ishihara M, Tadono T (2017). Land cover changes induced by the great east Japan earthquake in 2011. Sci. Rep..

[83] Focardi S, Materassi M, Innocenti G, Berzi D (2017). Kleptoparasitism and scavenging can stabilize ecosystem dynamics. Am. Nat..

[84] Osugi S, Trentin BE, Koike S (2019). Impact of wild boars on the feeding behavior of smaller frugivorous mammals. Mamm. Biol..

[85] Duľa M, Krofel M (2020). A cat in paradise: Hunting and feeding behaviour of Eurasian lynx among abundant naive prey. Mamm. Biol..

[86] Smith JB, Laatsch LJ, Beasley JC (2017). Spatial complexity of carcass location influences vertebrate scavenger efficiency and species composition. Sci. Rep..

[87] Moleón, M. *et al.* Carrion availability in space and time. In *Carrion Ecology and Management* (eds Olea, P.P., Mateo-Tomás, P. & Sánchez-Zapata, J.A.) 23–44 (Springer International Publishing, 2019).

[88] DeVault TL, Rhodes OE (2002). Identification of vertebrate scavengers of small mammal carcasses in a forested landscape. Acta Theriol..

[89] Bumann GB, Stauffer DF (2002). Scavenging of ruffed grouse in the Appalachians: Influences and implications. Wildl. Soc. Bull..

[90] Young A, Stillman R, Smith MJ, Korstjens AH (2014). An experimental study of vertebrate scavenging behavior in a Northwest European woodland context. J. Forensic Sci..

[91] Abernethy, E. F. *et al.* Carcasses of invasive species are predominantly utilized by invasive scavengers in an island ecosystem. *Ecosphere* **7** (2016).

[92] DeVault TL, Krochmal AR (2002). Scavenging by snakes: An examination of the literature. Herpetologica.

[93] Shivik JA, Clark L (1999). Ontogenetic shifts in carrion attractiveness to brown tree snakes (*Boiga irregularis*). J. Herpetol..

[94] Campobasso CP, Di Vella G, Introna F (2001). Factors affecting decomposition and Diptera colonization. Forensic Sci. Int..

